# Cross-Species Genome-Wide Analysis Reveals Molecular and Functional Diversity of the Unconventional Interferon-ω Subtype

**DOI:** 10.3389/fimmu.2019.01431

**Published:** 2019-06-25

**Authors:** Lauren E. Shields, Jordan Jennings, Qinfang Liu, Jinhwa Lee, Wenjun Ma, Frank Blecha, Laura C. Miller, Yongming Sang

**Affiliations:** ^1^Department of Agricultural and Environmental Sciences, College of Agriculture, Tennessee State University, Nashville, TN, United States; ^2^Department of Anatomy and Physiology, College of Veterinary Medicine, Kansas State University, Manhattan, KS, United States; ^3^Department of Diagnostic Medicine and Pathobiology, College of Veterinary Medicine, Kansas State University, Manhattan, KS, United States; ^4^Virus and Prion Diseases of Livestock Research Unit, National Animal Disease Center, USDA-ARS, Ames, IA, United States

**Keywords:** interferon, interferon-ω subtype, antiviral, molecular evolution, cytokine

## Abstract

Innate immune interferons (IFNs), particularly type I IFNs, are primary mediators regulating animal antiviral, antitumor, and cell-proliferative activity. These antiviral cytokines have evolved remarkable molecular and functional diversity to confront ever-evolving viral threats and physiological regulation. We have annotated IFN gene families across 110 animal genomes, and showed that IFN genes, after originating in jawed fishes, had several significant evolutionary surges in vertebrate species of amphibians, bats and ungulates, particularly pigs and cattle. For example, pigs have the largest but still expanding type I IFN family consisting of nearly 60 IFN-coding genes that encode seven IFN subtypes including multigene subtypes of IFN-α, -δ, and -ω. Whereas, subtypes such as IFN-α and -β have been widely studied in many species, the unconventional subtypes such as IFN-ω have barely been investigated. We have cross-species defined the IFN evolution, and shown that unconventional IFN subtypes particularly the IFN-ω subtype have evolved several novel features including: (1) being a signature multi-gene subtype expanding primarily in mammals such as bats and ungulates, (2) emerging isoforms that have superior antiviral potency than typical IFN-α, (3) highly cross-species antiviral (but little anti-proliferative) activity exerted in cells of humans and other mammalian species, and (4) demonstrating potential novel molecular and functional properties. This study focused on IFN-ω to investigate the immunogenetic evolution and functional diversity of unconventional IFN subtypes, which may further IFN-based novel antiviral design pertinent to their cross-species high antiviral and novel activities.

## Introduction

Innate immune interferons (IFNs), consisting of type I IFNs and type III IFNs, are key in regulating antiviral immunity, antitumor activity, and cell proliferation (1–6). In contrast to the single type II IFN (IFN-γ), which is primarily involved in adaptive immunity, type I IFNs are remarkable for their molecular and functional diversity. However, to date only a few subtypes (e.g., IFN-α and IFN-β) have been well characterized, even in humans and mice (1, 2, 4). Thus, there are significant knowledge gaps considering the evolutionarily diversified 20–60 IFN-coding genes of multiple subtypes in each mammalian species (2, 7–10). Interferon genes most likely emerged during tetrapod evolution from fish (2, 10–12). The common ancestor genes of IFNs were originally identified in jawed fish, which almost coincides with the emergence of animal adaptive immunity (2, 4). Fish only have a few ancestral intron-containing IFN genes, but more than a dozen IFN genes in each amniote species are mostly intronless (6–10, 12). The intronless type I IFNs in amniotes appear to have arisen from a retroposition event that is assumed to have replaced the original IFN locus by integration of intron-spliced RNA and, thus, favored subsequent gene duplication and family expansion adaptable to rapidly evolving viruses and functional divergence (8–11). We have examined IFN genes across the genome sequences of 120 animal species, and we specifically characterized the emergence and expansion of intronless IFNs in amphibians (10). In mammals, intronless type I IFNs have evolved through a subtype expansion resulting in at least nine subtypes, which include IFN-α, IFN-β, IFN-ε, IFN-κ, and IFN-ω commonly found in most mammalian species as well as IFN-δ (pigs), IFN-ζ (mice), and IFN-τ (cattle) only detected in some species. Moreover, subtypes including IFN-α, IFN-ω, IFN-δ, IFN-ζ, and IFN-τ have further diversified into multi-gene sub-clusters (7–12).

Pigs (and cattle) have the largest expansion of type I IFNs regarding subtypes and total IFN-coding gene numbers (8, 9). For example, porcine type I IFN gene loci contain 57 predictable IFN-coding genes (and 16 pseudogenes) spanning a nearly 1 Mbp genomic region and encoding at least 39 distinct IFN peptides assigned to 17 IFN-αs, 11 IFN-δs, 7 IFN-ωs, plus one each of IFN-β, IFN-ε, IFN-κ, and IFN-αω subtypes (9). Single IFN-ω-like genes are identified in reptiles and birds, and multi-gene IFN-ω subtypes are present in most ungulate and bat species. Through phylogenic analysis we showed that porcine IFN-ωs are orthologous to the majority of IFN-ω gene products identified so far in different species (9, 13). This further indicates porcine IFN-ω as a model to analyze functional novelty of this unconventional IFN subtype. Comparative genomic studies show that pigs have a molecular expansion of type I IFNs including IFN-ω genes, which are several-fold more than those in mice or humans (9, 13).

Whereas, subtypes such as IFN-α and IFN-β have been widely studied, the unconventional subtypes have been less investigated. In this study, we have determined IFN gene evolution across 120 animal genomes that are available in current genome resources, and shown evolutionary significance and gene expansion of IFN genes in several representative species of amphibians, bats, and ungulates. Further using the porcine and bovine IFN complexes as a model, we have determined the molecular and functional diversity of the evolving IFN subtypes, particularly the less-studied unconventional IFN-ω subtype, which is an IFN subtype emerging after reptiles and expanding in bats and ungulates (7–15).

Expansion of the IFN complex and IFN-ω diversity in bats and ungulates represent signature events of type I IFN evolution (7–15). We hypothesize that this subtype-expansion confers functional diversification that is necessary in regulating immune responses against species-specific and even cross-species viral infections (2, 7–15). Focusing on the IFN-ω members and their antiviral and inflammatory regulation, we characterized family-wide porcine innate immune IFNs for their functional spectrum and therapeutic potential (9, 10, 13, 14), which was profiled against two RNA viruses: porcine reproductive and respiratory syndrome virus (PRRSV), and influenza A virus. Both viruses have a high impact on the swine industry and influenza A virus in swine, also threatens public health (13, 16). Here, we show that vertebrate IFN-ω subtype has evolved several novel features, which include: (1) being a signature multi-gene subtype expanding particularly in bats and ungulates (7, 9, 17, 18), (2) emerging isoforms that have much higher antiviral potency than typical IFN-α (14, 18, 19), (3) highly cross-species antiviral (but little anti-proliferative) activity exerted in cells of humans and other mammalian species (20), and (4) other potential novel molecular and functional properties (3, 4, 18, 20). These observations suggest that animal unconventional IFN subtypes have diversified their molecular composition to extend functional spectrum particularly in antiviral and immunomodulatory regulation (1–11).

## Materials and Methods

### Ethics Statement, Animal Tissues, and Cells

Porcine tissues and primary cells used for gene expression and activity assays were cryopreserved samples from a previous study (9). The Institutional Biosafety and Institutional Animal Care and Use (IBC and IACUC) committees approved all recombinant DNA procedures and animal procedures, respectively. Samples of various tissues were collected, immediately snap frozen and stored in liquid nitrogen until used for nucleic acid or protein extraction (9, 13, 21).

Blood (~20 ml/pig) was collected by jugular venipuncture from anesthetized pigs. Immediately after euthanasia, cubes of ~0.5 cm^3^ were dissected from lung or other indicated tissues (9, 21, 22). Lungs were lavaged with 300 ml/each of 10 mM PBS (pH 7.4) (9, 13). Samples were placed on ice and peripheral blood mononuclear cells (PBMCs) and macrophages (M*ϕ*s) were isolated from the heparinized blood samples and lavage fluids, respectively, within 4 h after collection. PBMCs were isolated using a 60% Ficoll-Paque Plus gradient (GE Healthcare, Piscataway, NJ). Monocytes were isolated from the PBMCs with an anti-CD14 antibody (Ab), then the CD14^−^ population was used to isolate cDCs with a CD172 antibody (9, 13, 22), using magnetic beads conjugated with the corresponding secondary Ab (Miltenyi Biotec, Auburn, CA). The PRRSV-permissive mDCs were generated by culturing monocytes in the presence of IL-4 and GM-CSF for 7 days as described (9, 13, 22). Lavage fluid was centrifuged at 400× g for 15 min to collect cells and further isolate M*ϕ*s by plastic adherence (9, 22). Cells were used immediately or cryopreserved in Recovery™ cell culture freezing medium (Invitrogen, Carlsbad, CA).

Cell lines used for cell proliferation and antiviral assays were purchased from ATCC or transferred from collaborators at Kansas State University. Cells were cultured following ATCC's instruction or as previously described (10, 13). Cell lines used include porcine testis cells (ST, ATCC® CRL-1746™), porcine kidney cells (PK15, ATCC® CCL-33™), human intestinal epithelial cells (A549, ATCC® CRM-CCL-185™), mouse fibroblast cells (NIH/3T3, ATCC® CRL-1658™), mouse kidney cells (Mode-K), and the monkey kidney cells (MARC-145) (10, 13).

### Bioinformatics and Phylogenetic Procedures for Sequence Analyses

A combinative procedure was used to identify IFN genes (including both predictable IFN-coding genes and pseudogenes) in animal genomes (9, 10, 13). Single and grouped sequences, or hidden Markov model (HMM) profiles generated using sequence alignments with identified IFN peptide sequences in fish, birds, mammals, and amphibians, were used to query of genome assemblies available mainly through NCBI (http://www.ncbi.nlm.nih.gov/genome/80, and Ensembl (http://useast.ensembl.org/index.html), and sometimes through a species-concentrated database such as at Xenbase (http://www.xenbase.org/). Protein BLAST searches were conducted using the default algorithm parameters with BLOSUM62 matrix and Expected thresholds (E) <10 or 1. Resultant IFN homologs in each species were further used as query entries to inspect other more diversified IFN homologs in that species, which may generally have less pairwise identity (<40%) to cross-species IFN homologs.

The IFN genes were predicted and extracted from genomic sequences, which span the regions having translated frames significantly similar (~50% peptide similarity and E <10^−5^) to identified IFN sequences or consensuses. Programs interactively used for gene prediction include GenomeScan (http://hollywood.mit.edu/GENSCAN.html) and FGENESH (http://www.softberry.com), and were further manually annotated for confirmation. Peptide sequences were translated using the translate tool at the ExPASy port, and signal peptides were examined using PrediSi (http://www.predisi.de). Sequence alignments were generated primarily using the programs MUSCLE and ClustalW through EMBL-EBI port (http://www.ebi.ac.uk/), and other sequence management was conducted using programs at the Sequence Manipulation Suite (http://www.bioinformatics.org). Visualization of sequence alignments was conducted using Jalview, phylogenetic analyses using MEGA6, recombination analyses using SDT and RDP4, and topological comparison between the Newick trees was performed with Compare2Trees (http://www.mas.ncl.ac.uk/~ntmwn/compare2trees/). Other than indicated, all programs were run with default parameters (9, 10, 13).

### Gene Identification, Expression Analyses, and Cloning

Based on sequence analyses, we designed subtype-common or gene-specific primers for expression analyses using quantitative RT-PCR and cloning of coding regions from cDNA pools (Supplementary Material for primer sequence) (9, 13). For validation of the expression of various porcine IFNs, we amplified cDNA covering whole coding ORFs of representative genes in each gene or subgroup, cloned them in a pcDNA3.3 Topo-mammalian expression vector (Invitrogen, Carlsbad, CA), and confirmed them by sequencing. The cDNA was reverse transcribed from total RNA pools extracted from different tissues with a SuperScript III first-strand synthesis system and random primers (Invitrogen). Coding regions of IFNs were amplified from this cDNA pool for transcription confirmation and building expression constructs. Classification of porcine IFN stimulated genes (ISGs) into tunable or robust subgroups was referred to human or mouse ISGs, and gene-specific primers were designed and validated using porcine gene annotation (Supplemental Excel Sheet). PCR optimization, and real-time RT-PCR analysis were performed as described (9, 10, 13). In brief, gene-specific or subtype-common primers were designed based on multiple alignments of related IFN sequences, and PCR conditions were optimized and validated using confirmed IFN plasmids to show specific amplification only with templates containing confirmed IFN clone(s). RNA was extracted from tissues and cells as described above. Real-time RT-PCR assays were conducted in a 96-well microplate format using a StepOnePlus™ Real-Time PCR System (Applied Biosystems, Grand Island, NY) with the validated primers. Reactions were conducted with a SYBR Green RT-PCR system (Qiagen, Valencia, CA) with 100 ng of total RNA in a 20-μl reaction mixture. Specific optic detection was set at 78°C for 15 s after each amplification cycle of 95°C for 15 s, 56–59°C for 30 s, and 72°C for 40 s. Critical threshold (Ct) values and melt curves were monitored and collected with the real-time PCR system. Relative gene expression was first normalized against Ct values of the housekeeping gene (β-actin) for relative expression levels, and compared with the expression levels of control samples for stimulated regulation (9, 10, 13).

### Viruses

All experiments using infectious viruses were conducted in the laboratories with licenses and handled according to restrictive regulations specified. Viruses used in this study tested include a PRRSV P129 strain, and influenza A virus strains including 2009 pandemic H1N1 A/CA/04/09 (pH1N1), H1N1A/WSN/1933 (WSN), and H1N1A/swine/Kansas/77778/2007 (KS07). All experiments using infectious agents were conducted in the laboratories covered by effective licenses and handled according to restrictive regulations specified. Viruses tested include a PRRSV P129 strain, and influenza strains of A/(H1N1)/pdm09 (pH1N1), A(H1N1)/WSN/1933(WSN), and A(H1N1)/swine/Kansas/77778/2007(KS07). Procedures for viral infections were conducted in cells as previously described (9, 10, 13). Cytopathic effect (CPE) and immunochemical staining of viruses were used to measure viral infectivity and titers.

### Antiviral Activity

IFN peptides were expressed using two eukaryotic systems, a HEK293-mammalian expression system (Invitrogen) and a yeast expression system through collaboration with Kingfisher Biotech (St. Paul, MN). The molecular authenticity of IFN peptides expressed by both systems was verified with the following aspects: (1) gene sequences; (2) protein band pattern on protein gels; and (3) potential antiviral function tested in different cell-virus systems. It is noteworthy that IFN peptides expressed by both systems have been comparatively determined multiple times to demonstrate the duplicity as shown in several publications (9, 13, 14, 23, 24). The antiviral activity of IFN peptides was tested on the cell/virus systems including: MARC-145/PRRSV, porcine MΦ/PRRSV, A549/pH1N1, PK-15/pH1N1, Mode-K/WSN, NIH3T3/WSN, Mode-K/KS07, and NIH3T3/KS07, respectively. Briefly, cells were seeded in flat-bottom 96-well plates and grown to >95% confluence. Cells were infected with PRRSV or influenza viruses as indicated, and treated with 1:10 serially diluted IFN peptides at 20–2 × 10^−10^ ng/ml. Viral infection was determined by staining the cytopathic effect on cell monolayer with 1% crystal violet or immuno-staining of the viruses, and quantified with a SpectraMax i3 (Molecular Devices) spectrometer. Antiviral activity of IFNs was calculated using the Reed–Muench Method to normalize TCID_50_ and expressed as U/μg/ml. One unit (U) is the highest dilution that reduced cell number by 50% (9, 10, 13).

### Acidic and Thermal Stability

Interferon peptides were incubated at pH 2 for 24 h at 4°C, or at 42, 56, or 63°C for 5 h, as described previously (25), and the remaining antiviral activities of the treated and untreated samples were then compared using the MARC-145/PRRSV system as described above (9, 10, 13).

### Bioassays and ELISA

IFN bioassays were conducted in MARC-145 cells stably transformed with IRF3-, IRF7-, or Mx1-promoter driven luciferase reporter systems (9, 13, 22). In brief, for bioassays, MARC-145 (IRF3, IRF7, or Mx1) cells were treated with IFN peptides at indicated concentrations for 24 h, lysed with Glo lysis buffer and quantified by Steady-Glo® Luciferase Assay System (Promega) (13, 23).

### Anti-proliferative Activity Assay

Cellular viability was assessed by trypan blue dye exclusion (Invitrogen, Carlsbad, CA). Five-hundred cells in 100 μL volume were added to each well of a flat-bottom 96-well plate in triplicate. Cells were treated with or without IFN peptides at the indicated concentrations (1:10 serially diluted from 2 ng/ml) and incubated at 37°C for 72 h. Cell growth was determined by a MTS-salt [3-(4,5-dimethythiazol-2-yl)]-5-(3-carboxymethoxyphenyl)-2-(4-sulfophenyl)-2H-tetrazolium) assay (Promega, Madison, WI). Briefly, 20 μl of MTS reagent were added to each well and the plate incubated for 2–4 h at 37°C. Following color development, absorbance was measured at 490 nm on a SpectraMax i3 (Molecular Devices) spectrometer (23, 26).

### Statistical Analysis

All statistical analyses were performed using Student's *t*-test. Data are presented as mean ± SEM. A *p* value of < 0.05 was considered statistically significant (9, 10, 13, 23).

## Results

### Cross-Species IFN Gene Annotation and Evolutionary Determination

**Figure 1:**
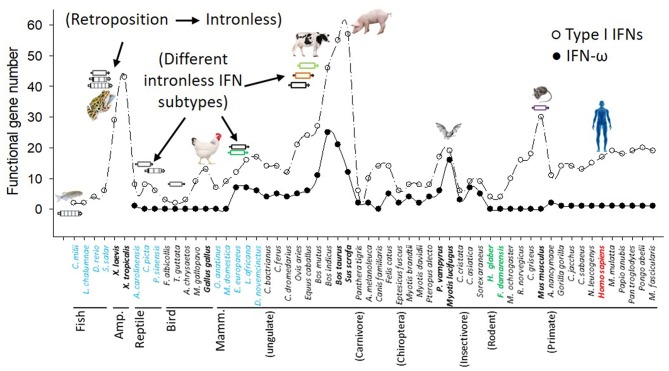
Molecular evolution and diversification of type I IFNs and IFN-ω subtype in representative vertebrate species. Functional IFN gene numbers are annotated from released genomes of representative species and plotted along the phylogenetic order according to NCBI Taxonomy at http://www.ncbi.nlm.nih.gov/Taxonomy. Several major events including the retroposition leading to emergence of intronless IFNs in amphibians, and expansion of IFNs in amphibians, livestock, bats, and mice are shown (8–10).

Innate immune IFNs most likely emerged during tetrapod evolution from fish (2, 5, 9, 10, 12). Cross-species genome-wide annotation verified that fish only have a few ancestral intron-containing IFNs; however, multiple IFN genes in each amniote species are mostly intronless (2, 5, 9–12). The intronless type I IFNs in amniotes appear to have arisen from a retroposition event that is assumed to have replaced the original IFN locus by the integration of intron-spliced RNA and, thus, favored subsequent gene duplication and family expansion adaptable to rapidly evolving viruses and functional divergence (2, 9–12). We have genome-wide examined IFN genes across the genome sequences of nearly 120 representative species of vertebrates, and identified the emergence and expansion of intronless IFNs in amphibians (10–12). For example, in two *Xenopus* genomes, 13–16 intron-containing IFN genes (of both type I and type III IFNs) exist that retain intron-containing gene structure as fish IFN genes, and 24–37 intronless IFN genes, indicating the emergence and expansion of intronless IFN genes in amphibians rather than in reptiles as previously assumed (Figure 1 and Supplemental Excel Sheet) (10–12). Although type I IFN genes kept evolving to be intronless in reptiles and birds, the gene diversification process became less active than in amphibians, as most reptile and bird species contain several IFN genes similar to those in the fish but they are mostly, or nearly intronless (2, 10–12). Among the analyzed bird species, the domestic chicken and duck have the most 9–10 IFN-coding genes. Dramatic IFN gene diversification further occurred in several mammalian species. With regard to IFN-coding genes, ungulate species such as cattle (*Bos taurus/indicus*) and pigs (*Sus scrofa*) have nearly 60 predictable IFN-coding genes as well as more than a dozen pseudogenes (8, 9, 12). Other mammalian species show IFN gene expansion with more than 20 predictable IFN-coding genes including the ungulates domestic sheep, horses, and yak, the house mouse and many primate species including human and pongo (7). Most other wild mammalian species generally have 7–16 predictable IFN-coding genes, except two underground-living mole rats (*H. glaber* and *F. damarensis*) that have most redundant compositions of type I IFN coding genes comparable to fish. In summary, cross-species and genome-wide definition of IFN genes in vertebrate species determined previously unknown molecular complexity of IFN expansion in Amphibian (10), Chiroptera, Rodent (except the two moles) and domestic ungulate species especially pigs and cattle (2, 8–10). This revises the linear-increasing pattern of IFN molecular evolution as previously proposed along amniotic evolution (12). Several gene expanding-surges are particularly evident in amphibians, domestic birds and ungulates, as well as some rodent species, which illustrates a lineage, even species-independent “bouncing model” with multiple peaks rather than the previous linear-increase-model accompanying amniotic evolution (Figure 1) (2, 10, 12).

### The Specification of IFN-ω Subtype During the Evolution of IFN Subtypes

The archetypal subtypes of type I IFNs in mammals such as IFN-β, -ε, and –κ, appear ramified in reptiles, and IFN-α and –ω subtypes were remarkable in birds with some ambiguous progenitors detected in reptiles, as well (Supplemental Excel Sheet 2) (2, 12). In contrast, the gene composition of type III IFNs (i.e., IFN-λs) was ramified and expanded in amphibians; however it was reduced dramatically in reptiles and birds (generally only one IFN-λ gene) and remained relatively stable (generally 3–5) in different mammalian clades/species (5, 9). In addition, typical intronless IFN-λ genes were only determined in amphibian species, and rarely determined in mammalian species except a bat species (*Myotis brandtii*) (Supplemental Excel Sheet 2 and Data not shown).

All typical subtypes of type I IFNs were diversified in different clades of mammalian species. In mammals, intronless type I IFNs have evolved through a subtype expansion resulting in at least nine subtypes, which include IFN-α, IFN-β, IFN-ε, IFN-κ, and IFN-ω commonly found in most mammalian species as well as IFN-δ (pigs), IFN-ζ (mice), IFN-τ (cattle), and IFN-αω (or –μ) (pigs, horses, and cattle) only detected in some species (7–9, 27). Moreover, subtypes including IFN-α, IFN-ω, IFN-δ, IFN-ζ, and IFN-τ have further diversified into multi-gene sub-clusters (7–9, 27). In terms of subtypes and total functional gene numbers, pigs (and cattle) have the largest expansion of type I IFNs (7–9) (Figure 1). For example, porcine type I IFN gene loci contain 57 predictable IFN-coding genes (and 16 pseudogenes) spanning a nearly 1 Mbp genomic region and encoding at least 39 distinct IFN peptides assigned to 17 IFN-αs, 11 IFN-δs, 7 IFN-ωs, plus one each of IFN-β, IFN-ε, IFN-κ, and IFN-αω subtypes (7–9, 13).

**Figure 2:**
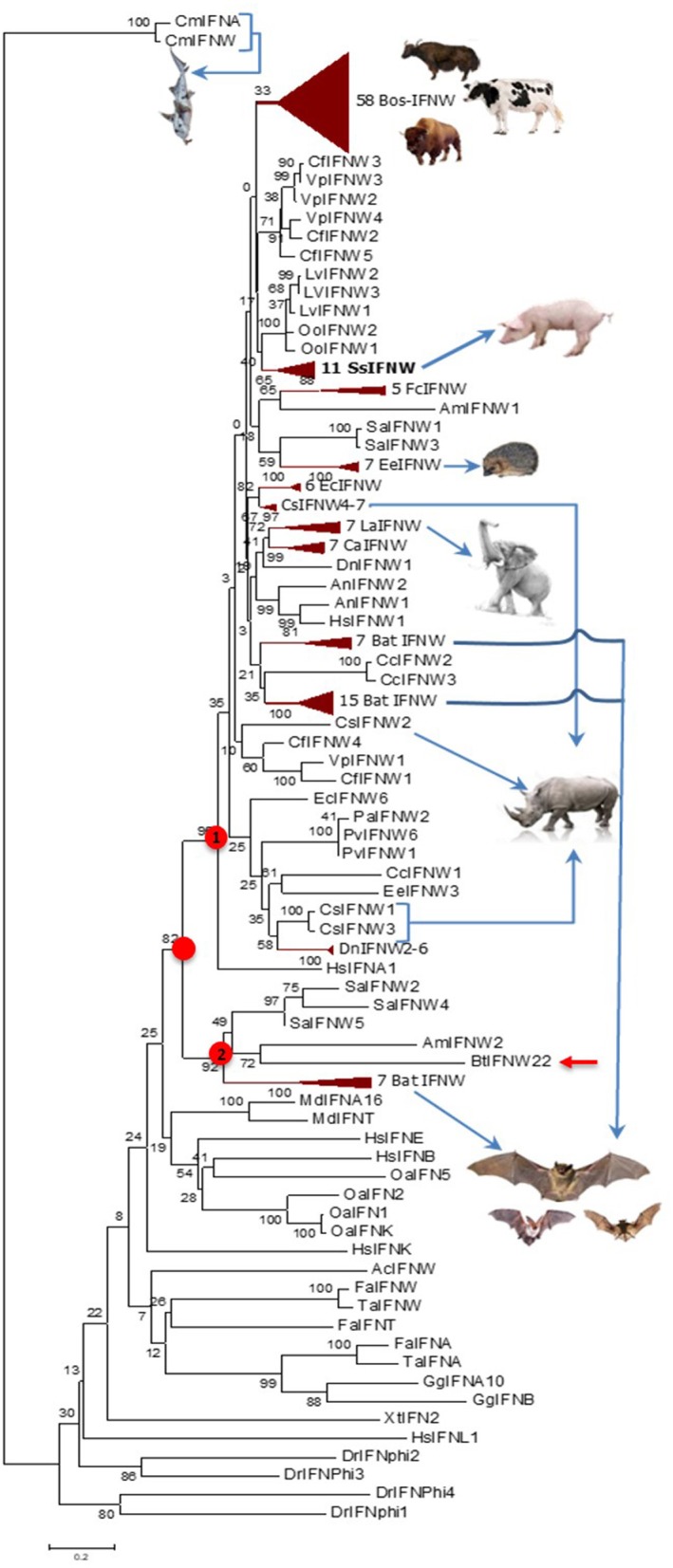
Evolutionary relationships of the IFN-ω orthologs in different animal species/lineage, and comparison with homologs from zebrafish (DrIFNs), Xenopus (XtIFNs), chicken (GgIFNs), and humans (HsIFNs). The evolutionary history was inferred using the Neighbor-Joining method. Percentage of replicate trees in which the associated taxa clustered together in the bootstrap test (1,000 replicates) is shown next to the branches. The tree is drawn to scale, with branch lengths in the same units as those of the evolutionary distances used to infer the phylogenetic tree. The evolutionary distances were computed using the p-distance method and are in units of the number of amino acid differences per site. Evolutionary analyses were conducted in MEGA6. The IFN-ω subtype diversified at the similar time of IFN-α, IFN-β, or IFN-ε subtypes for the IFN ancestral molecules identified in jawed fish, but independently evolve further particularly in different mammalian lineage/species. Most, if not all, IFN-ω orthologs form into a big cluster that further bifurcate into two subclusters (labeled with red dots), and the subcluster 1 comprises a majority of IFN-ω orthologs. IFN taxa used: IFNA, IFNB, IFNE, IFNK, IFNL, and IFNW correspond to genes for IFN-α, IFN-β, IFN-ε, IFN-κ, IFN-λ, and IFN-ω, respectively, in classic nomenclature, and stand for relevant IFN protein precursors here (2, 8–10).

Cross-species examination of IFN-ω genes indicated that single IFN-ω-like genes are identified in reptiles and birds, and multi-gene IFN-ω subtype are present in most ungulate and bat species (Figures 1, 2, and Supplemental Excel Sheet). Through phylogenic analysis, we showed that different species of ungulates particularly domestic species such as cattle and pigs experienced individual IFN-ω expansion because IFN-ω genes of each species generally forming into one or two major expanding clusters (2, 8, 9). Compared to generally one to several IFN-ω genes in other species, cattle and pigs may have 11–25 IFN-ω coding genes as well as about a dozen pseudogenes (Figure 2) (8, 9). Porcine IFN-ω isoforms are orthologous to most, if not all, IFN-ω gene products identified in mammalian species and particularly the complex in cattle (7–9, 17, 18, 27). This further identifies porcine IFN-ω as a model to analyze functional novelty of this unconventional IFN subtype (Figures 1, 2) (7–9, 13). Thus, comparative genomic studies show that pigs and cattle are excellent examples demonstrating a molecular expansion of type I IFNs including IFN-ω genes, which are several-fold more than those in mice or humans (Figures 1, 2) (7–9, 17, 18, 27).

### Genetic Polymorphisms of IFN-ω Genes

**Figure 3:**
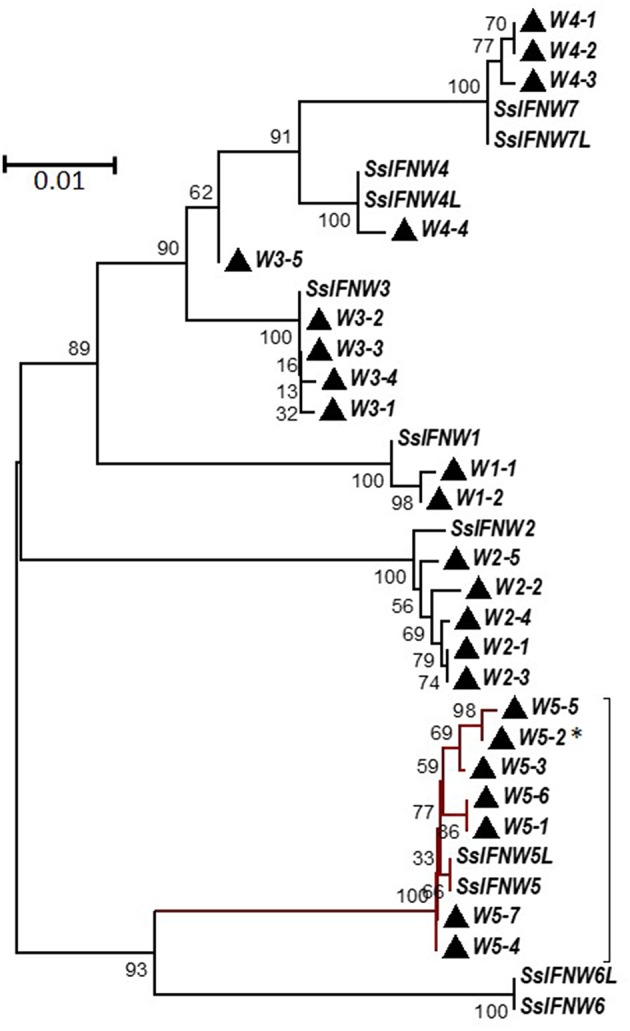
Phylogenetic relationships of porcine IFN-ω genes (SsIFNW1-7) and identified genomic polymorphisms (Ws). SsIFNW1-7 represent seven original identifications of IFN-ω genes, of which they share <95% sequence identity among each other. SsIFNW4L, 5L, 6L, and 7L are nearly identical duplicates of SsIFNW4-7, respectively; they are further annotated from current swine genome assembly (Sscorfa10.2). All genomic polymorphisms generally have only one to several nucleotide mutations and are clustered with their parental SsIFNW genes in the same branches. Note, more SsIFNW5 polymorphisms have been identified, and they exert diverse antiviral activity as partly shown in **Figure 9**. The associated taxa clustered together in the bootstrap test (1,000 replicates) are shown next to the branches. Evolutionary analyses were conducted with MEGA6 (http://www.megasoftware.net/). ^*^Primary IFN-W5 sequence used for activity assay.

Genetic polymorphisms of a few nucleotide residues are frequently found in the promoter or coding regions of type I IFN genes (14, 28, 29). We and others have found that striking differences in IFN activity is associated with simple polymorphic mutations (14, 28, 29). This implies a genomic mechanism of IFN-system evolution, which is critical in the arms race with ever-mutating viruses to create a novel antiviral genotype. Regarding porcine IFN-ω genes, on the basis of the 11 porcine IFN-ω coding genes determined in the reference swine genome assembly (Sscrofa11.1, NCBI), extensive sequencing of IFN genes isolated from the DNA pool of 400 pig blood samples allowed us to identify 3–7 SNP of each IFN-ω functional gene, with the IFN-ω5 gene having the maximal 7 SNP identified in that DNA pool (14). These several porcine IFN-ω5 polymorphic isoforms (Figure 3), which only differ from each other by few residues, showed dramatic activity differences (**Figures 6**-**9**; next) (9, 13, 14, 24).

### Subgroups of Vertebrate IFN-ω Peptides

**Figure 4:**
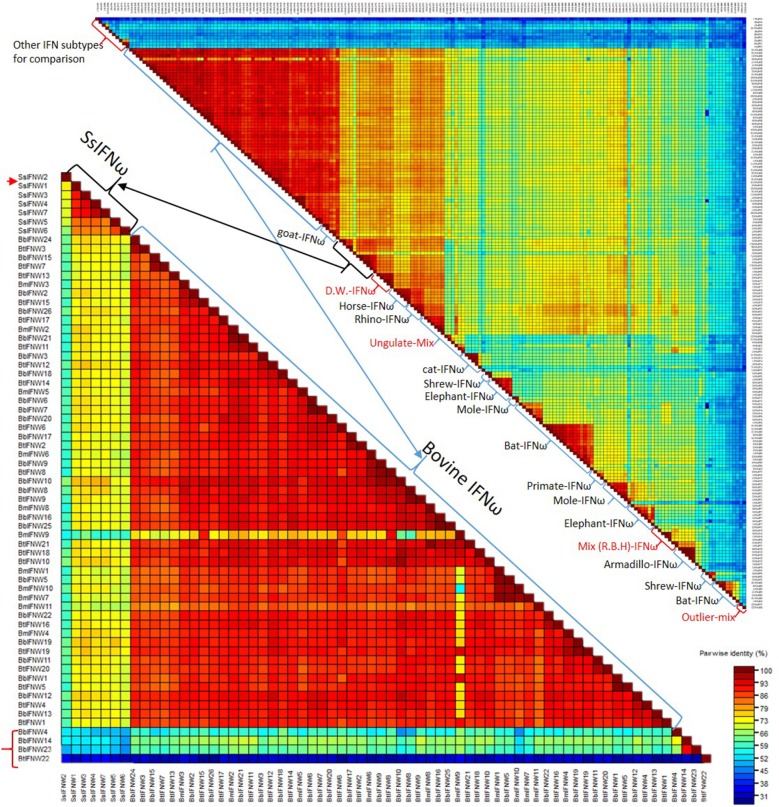
Pairwise identity (%) plots among protein sequences of IFN-ω orthologs. Comparison and plot drawing were performed using a SDT program. IFN-ω molecules from individual Order or Genus might share a common progenitor, as also shown in the Figure 2 for the clustered phylogenic clades that share >86% of identity. It was also common to observe that IFN-ω molecule from a species is phylogenically closer to the orthologs from other species of the same Family/Genus than those from the same species. For the mammalian genus/species that have multiple genes of IFN-ω subtype, we observed that IFN-ω peptides such as in bats, moles, shrews and elephants are formed into two major sub-clusters (**Upper**); however, it is primarily only one sub-cluster (with one to several “outliers,” labeled with red arrow or brackets) such as in swine and bovine species (**Bottom**). In addition, several clusters of IFN-ω peptides contain IFN-ω peptides from animals of different Genus/Family, such as that of the ungulate-mix (Alpaca, Camel, and Bioson) and the RBH-mix (Rhino, Bat, and Horse) clusters (**Upper**). RBH: R. Rhino, B. Bat, and H, horse; and other Species name abbreviations are first letters from the Latin names listed in the Supplemental Excel Sheet according to NCBI Taxonomy at http://www.ncbi.nlm.nih.gov/Taxonomy (2, 8–10).

Figure 4 shows pairwise identity (%) plots among protein sequences of all identified IFN-ω orthologs across genome sequences of about 60 representative vertebrate species (Figure 4, upper panel, and Supplemental Excel Sheet) and the expanding IFN-ω paralogs in swine and bovine species (Figure 4, bottom panel, and Supplemental Excel Sheet) (7–9, 17, 18, 27). Generally, we detected segregation of IFN-ω subtype in birds, but the definitive formation of multi-gene IFN-ω was detected in the genomes of mammals from all Orders except Monotremes, Marsupials, and Rodents. Further phylogenic analysis indicated that IFN-ω molecules from individual Order or Genus might share one common progenitor, as shown in Figure 2 for the clustered phylogenic clades and in Figure 4 for peptides that share >86% of identity. It was common to observe that IFN-ω molecules from one species are phylogenically closer to the orthologs from other species of the same Family/Genus than those from the same species (Figures 2, 4). For the mammalian genus/species that have multiple genes of IFN-ω subtype, we observed that IFN-ω peptides such as in bats, moles, shrews, and elephants are formed into two major sub-clusters; however, it is primarily only one sub-cluster (with one to several “outliers”) such as in swine and bovine species (Figures 2, 4). In addition, several clusters of IFN-ω peptides contain IFN-ω peptides from animals of different Genus/Family, such as that of the ungulate-mix (alpaca, camel, and bioson) and the RBH-mix (rhino, bat, and horse) clusters (Figure 4, upper panel). In contrast to the potential evolutionary progenitor shared in mammalian species, IFN-ω genes undergo reduction or expansion independently in each animal species, which are especially evident, such as IFN-ω gene expansion in swine and bovine species (Figure 4, bottom panel).

### Constitutive and Induced Expression of Porcine IFN Genes

**Figure 5:**
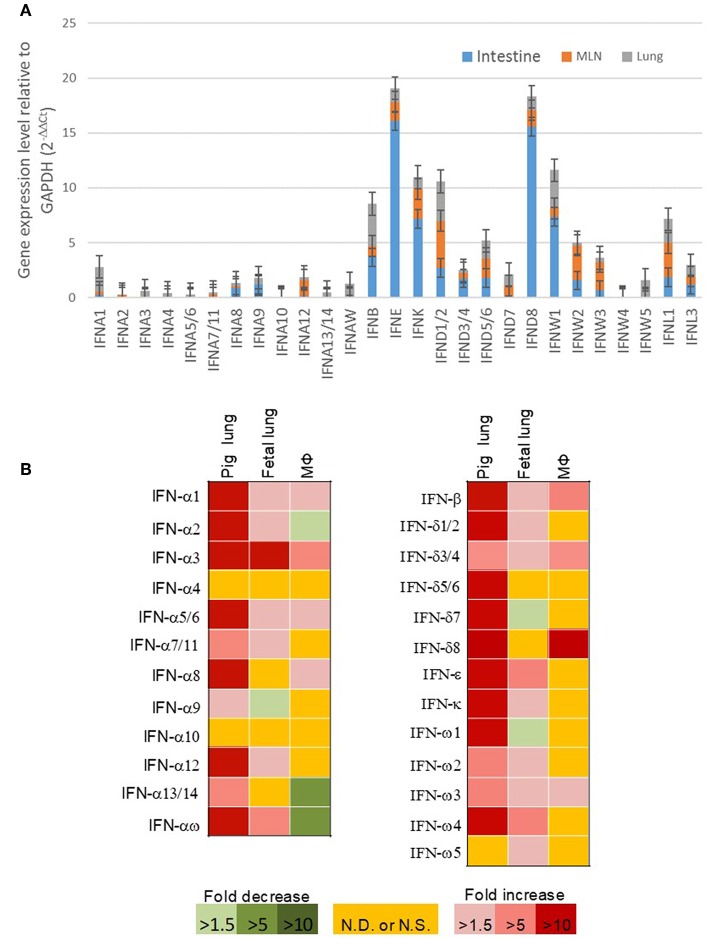
**(A)** Relative gene expression of porcine type I IFNs in the intestine, mesenteric lymph nodes, and lung from normal 5-week-old pigs. Real time RT-PCR assays were conducted as in Manry et al. (7), basal expression of IFN genes was normalized against Ct values of a housekeeping gene (GAPDH) and presented as relative expression index. Compared with the genes of IFN-α subtype (gene symbol IFNA, and so on) that are mostly inductive, unconventional porcine type I IFN genes including IFNWs show much higher constitutive expression in mucosal tissues and lymph nodes. Data, *n* = 3 PCR repeats of tissue samples from 3 to 5 pigs. **(B)** Sub-optimal stimulation of porcine type I IFNs in PRRSV-exposed fetal lungs and alveolar macrophages (M*Φ*). Lungs of fetuses and pregnant sows infected with PRRSV at the 90th of the gestation day, and porcine alveolar M*Φ* infected *in vitro* with PRRSV for 6 h were evaluated for mRNA expression of IFNs. Data, *n* = 3 PCR repeats of tissue samples from 3 samples (9, 10, 13, 24).

Our previous expression analysis of porcine type I IFNs in normal intestine, lymph nodes, and lung revealed that epithelial and constitutive expression of unconventional IFNs (particularly IFN-ε, -κ, -δ, and -ω) in contrast to IFN-α subtype that is prone to an inductive expression during antiviral responses (9, 13). Among IFN-ω subtype, IFN-ω1, -ω2, and -ω3 were expressed higher than IFN-ω4 and –ω5 (Figure 5A). In response to the viral infection, we demonstrated that PRRSV-infection induced higher expression (5–10-fold than the control) of multiple IFN subtypes/genes in the lungs from adult sows that had been infected for 14 days. However, the induced expression of IFNs was much weaker (<5-fold) and even suppressed by the PRRSV infection in the fetus from the PRRSV-infected sows and especially unresponsive in the alveolar macrophages infected for 6 h *in vitro* (Figure 5B). Collectively, these findings show that innate immune IFN expression is not restricted to antiviral responses but is extensively involved in immune homeostatic regulation in epithelial mucosa, where inflammation is restricted for normal physiological functions (9, 13, 30–33).

### Higher Acidic- and Thermal Stability of Porcine IFN-ω Subtype

Compared with IFN-γ, two major physiochemical property of type I IFN peptides are their tolerance in acidic solutions (pH < 4.5), but less thermal stability in higher temperature (1, 20, 25). For comparative activity assays, we synthesized several porcine IFN peptides using both a mammalian expression system (HEK293F, Invitrogen) for authentic verification at small scale (9, 13) and a yeast-expression system (Kingfisher Biotech) for bulk production (24). Pertaining to antiviral activity compared in several virus-cell systems (conducted both previously and in this study), IFN peptides produced in both mammalian and yeast system were very comparable and exerted very similar antiviral activity (9, 24). We determined the acidic and thermal stability of IFN-ω1 and IFN-ω5 peptides in comparison to IFN-α1 peptide. As shown in Table 1, both IFN-α and IFN-ω peptides had similar acidic stability after incubation with an acetic acid buffer at pH 2.0 for 24 h at 4°C. Incubation of IFN peptides in acidic buffer caused almost no loss in antiviral activity against PRRSV infection in MARC-145 cells (Table 1). In contrast, high temperatures at 42, 56, or 63°C for 5 h removed all activity of the IFN-α peptide; however, IFN-ω peptides showed better thermal stability. IFN-ω1 retained most active after treated at 42°C, IFN-ω5 retained much activity even when treated at 56 or 63°C for 5 h (Table 1). We also examined the acidic and thermal stability using a more sensitive ISG-promoter reporter luciferase assay and observed similar results as that of the antiviral assay (data not shown) (13, 23). Hence, porcine IFN-ω peptides exert better thermal stability but similar acidic stability compared to IFN-α peptide (20, 25).

**Table 1:** Higher acidic- and heat-stability of porcine IFN-ω subtype.

	**IFN concentrations (μg/ml) reduced cell loss by 50% according to Reed-Muench method**				
	**Untreated**	**pH = 2.0**	**42^**°**^C**	**56^**°**^C**	**63^**°**^C**
IFN-α1	1.1 × 10^−3^	1.1 × 10^−3^	-	-	N
IFN-ω1	1.1 × 10^−3^	1.1 × 10^−3^	2.0 × 10^−2^^*^	-	-
IFN-ω5	1.1 × 10^−5^	1.1 × 10^−5^	1.1 × 10^−5^	1.1 × 10^−3^^*^	1.1 × 10^−2^^*^

*N, no test; -, no antiviral activity with the tested IFN concentrations as high as 0.2 μg/ml after the treatments*.

* *p < 0.05; compared to untreated control*.

### Anti-proliferative Activity in Cells From Different Animal Species

**Figure 6:**
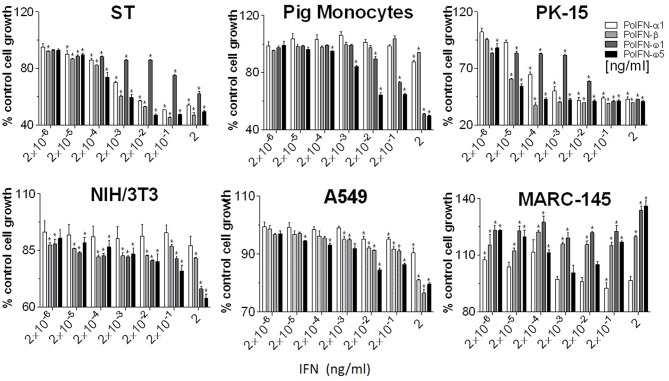
Interferon antiproliferative activity assay. Cellular viability was assessed by trypan blue dye exclusion (23, 24). Five-hundred cells in 100 μl volume were added to each well of a flat-bottom 96-well plate in triplicate. Cells were treated with or without IFN peptides at indicated concentrations and incubated at 37°C for 72 h. Cell growth was determined by a MTS-salt assay (Promega, Madison, WI). Briefly, 20 μl of MTS reagent was added to each well and the plate incubated for 2–4 h at 37°C. Following color development, absorbance was measured at 490 nm on a with a SpectraMax i3 (Molecular Devices) spectrometer. Data, mean ± SE, *n* = 6, ^*^p < 0.05 compared to the mock-treated control.

Anti-proliferative activity underlies anti-tumor mechanism of IFNs (4, 34). Compared with classical IFN-α1 and IFN-β, porcine IFN-ω1 and -ω5 (at the concentrations > 0.02 ng/ml) displayed higher antiproliferative activity in porcine monocytes. In porcine epithelial cell lines from testes (ST) or kidney (PK-15), IFN-ω5 but not IFN-ω1 exerted similar antiproliferative activity as IFN-α1 and IFN-β at the tested concentrations (Figure 6, top panel). Surprisingly, porcine IFN-ω1 and IFN-ω5 also significantly suppressed the proliferation of mouse cell lines, but actually showed stimulation of cell proliferation in both human and monkey kidney cells (A549 and MARC-145). Collectively, porcine IFN-ω subtype, in particular the high antiviral IFN-ω5 also exerted higher antiproliferative activity in all tested porcine cells and mouse cells, but no activity in tested primate cells.

### Induction of Interferon-Stimulated Genes (ISGs)

**Figure 7:**
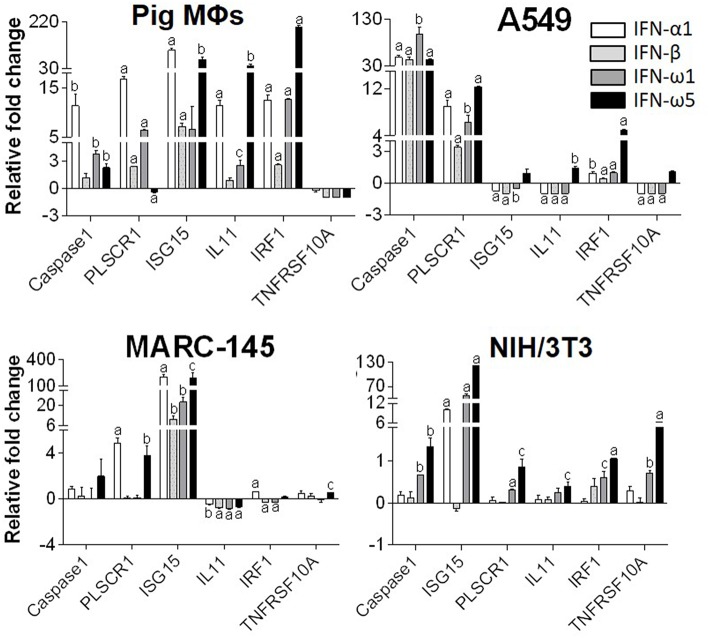
Differential induction of IFN-stimulated genes (ISGs) by porcine IFN peptides in porcine (M*Φ*s), monkey (MARC-145), human (A549), and mouse (NIH3T3) cells. Cells at 80% confluence were treated with overexpressed IFN peptides (20 ng/ml) for 24 h. Gene expression was analyzed using a SYBR Green-based real-time RT-PCR assay. Total RNA (100 ng) was used in each 20 μl of PCR reaction. Ct values of the genes were normalized against Ct values of a housekeeping gene (beta-actin) amplified from the same RNA samples to obtain 2^−Δ*Ct*^, which reflects the expression of each ISG relative to beta-actin and were further normalized for fold changes to the control (mock). CASPAS1, caspase 1; IL11, interleukin 11; IRF1, IFN-regulatory factor 1; ISG15, ubiquitin-like IFN stimulated gene 15; PLSCR1, phospholipid scramblase 1; TNFRSF10A, tumor necrosis factor alpha receptor superfamily 10A (10, 24). Data are means ± SE; *n* = 3 replicates of 2–3 independent assays, a, b, and c: *p* < 0.001, 0.01, and 0.05, respectively, compared to control.

**Figure 8:**
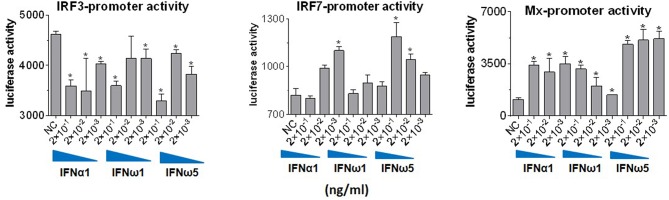
IFN comparative activity bioassay in MARC-145 cells that stably transformed with an IFN-regulatory factor (IRF)3-, IRF7-, or a myxovirus resistance gene 1 (Mx1)-promoter driven luciferase reporter system. MARC-145 cells that were transformed with the promoter-reporter constructs were treated with IFN peptides at indicated concentrations for 24 h, lysed with Glo lysis buffer and quantified by Steady-Glo® Luciferase Assay System (Promega) (13, 24). Data are means ± SE; *n* = 3; ^*^p < 0.05 relative to the control.

Innate immune IFNs confer antiviral and immunomodulatory roles through induction of hundreds of IFN stimulated genes (ISGs), which are generally classified into robust or tunable ISGs relative to their responsive intensity to IFN stimulation (10, 35, 36). Whereas, most robust ISGs are involved in antiviral responses, tunable ISGs are more broadly modulatory for immune and developmental regulation (35, 36). To test the differential potency of porcine IFN-ω in induction of ISGs, we measured the expression of six typical ISGs (three robust and three tunable) (10, 35, 36) in animal and human cells treated with the overexpressed peptides (IFN-α1, IFN-β, IFN-ω1, and IFN-ω5) for 24 h. The three robust ISGs are caspase 1, phospholipid scramblase 1 (PLSCR1), and ubiquitin-like ISG15 (ISG15); and the three tunable ISGs include interleukin 11 (IL11), IFN-regulatory factor 1 (IRF1), and tumor necrosis factor alpha receptor superfamily 10A (TNFRSF10A). Data show that, IFN-ω1 and IFN-ω5 stimulated the robust ISGs (esp. ISG15) to an extent similar to or higher than IFN-β and IFN-α1, respectively, in porcine or non-porcine cells. However, IFN-β and IFN-α1 are generally less active than IFN-ω5 in stimulation of the three tunable ISGs in the cells of non-porcine origin (Figure 7). Interferon-regulatory factor (IRF) 3, IRF7 and Myxovirus resistance protein 1 (Mx1) genes are other representative ISGs that play key roles in IFN auto-regulation (such as IRF3 and IRF7 in further potentiation of IFN-β and IFN-α production in macrophages and pDCs, respectively) and in anti-Myxovirus (such as influenza) activity (10, 35, 36). Using a promoter-reporter based bioassay (13, 23), we analyzed the stimulation of IRF3, IRF7, and Mx1 expression by treatment with different concentration of IFN-α1, IFN-ω1, and IFN-ω5. Porcine IFN-ωs, particularly IFN-ω5 exerted higher activity in stimulation of IRF7 and Mx1 expression, but are similar to IFN-α1 for the effect on IRF3 promoter (Figure 8). In summary, porcine IFN-ω subtypes, especially the highly antiviral IFN-ω5, potentially signal ISG expression to exert antiviral immunity differently from the classical IFN-α1 and IFN-β subtypes in both porcine and human cells (13, 23).

### Broad and Higher Antiviral Activity

**Figure 9:**
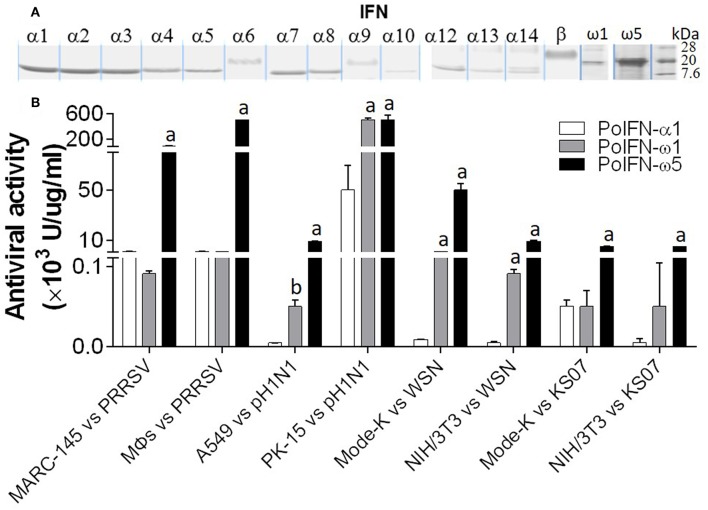
Porcine IFN-ω peptides exert broad and high antiviral activity in porcine, monkey, human, and mouse cells. **(A)** A series of porcine IFN peptides were overexpressed using a mammalian cell expression system (HEK193F, Invitrogen, Carlsbad, CA). IFN peptides were collected and partially purified using two Centricon® centrifugal filters (10 and 50 k NMWL, Millipore, Billerica, MA). Concentrated IFN-peptides were subjected to gel electrophoresis and stained with a bio-safe Coomassie Blue G-250 solution. Shown are peptide bands of most IFN-α/β subtypes, IFN-ω1, and IFN-ω5. Note that the bands of IFN-α6, IFN-α9, and IFN-β are smeared and have apparent molecular weights higher than expected values (~20 kDa), which might reflect posttranslational modification such as N-glycosylation (13, 23). **(B)** Porcine IFN-ω peptides exert broad and high antiviral activity against PRRSV and influenza A virus in cells from pigs, monkeys, humans, and mice. The antiviral activity of IFN peptides was titrated as described (7), except viruses were quantified using fluorescence-labeling (PRRSV-DsRed and FITC-immunostaining for influenza A nucleoprotein) in the susceptible cells of monkey MARC-145, porcine macrophages (M*Φ*s) and PK-15, human A549, and mouse Mode-K and NIH3T3 (13, 24). A PRRSV-P129 strain, and three influenza strains, pH1N1, WSN, and KS07 were used. a,b; *p* < 0.001, 0.01 to IFN-α1, respectively. Data are means ± SE; *n* = 5.

We have compared the antiviral activity of 20 porcine IFN peptides family-wide. Compared with typical subtypes of porcine type I and III IFNs including IFN-α and -β, IFN-ω exerted most broad antiviral activity. Using VSV and PRRSV, we previously reported that in porcine and monkey cells, most IFN-α peptides showed high antiviral activity on average (9, 13). Interestingly, IFN-ω peptides exert broad antiviral activity including IFN-ω1 to IFN-ω2 having generally low to mid antiviral activity, and IFN-ω5 (particularly, polymorphic mutant of IFNω5-2 in Figure 3) exerting the highest antiviral activity. Other subtypes, including IFN-β and most IFN-δ induced much lower antiviral activity (9, 13). In Figure 9, we further compared antiviral activity of porcine IFN-α1, IFN-ω1, and IFN-ω5 against PRRSV (a type II P129 strain) and several strains of influenza A virus (pH1N1, WSN, and K07 strain) in corresponding porcine, mouse and primate cells that are susceptible to the indicated virus infection. IFN-α1 generally had an activity at 10^3^-10^4^ U/μg/ml against PRRSV in porcine and MARC-145 cells, but had little activity against influenza viruses in human and mouse cells. In contrast, IFN-ω1 and particularly IFN-ω5, were broadly and highly active against both PRRSV and influenza viruses in cells from the four mammalian species. In all eight types of virus-cell infection systems, IFN-ω5 exerted the highest antiviral activity (100–1,000-fold higher than IFN-α1) for PRRSV and influenza viruses (Figure 9).

### The “Outliers” of the Unconventional IFN Subtypes

**Figure 10:**
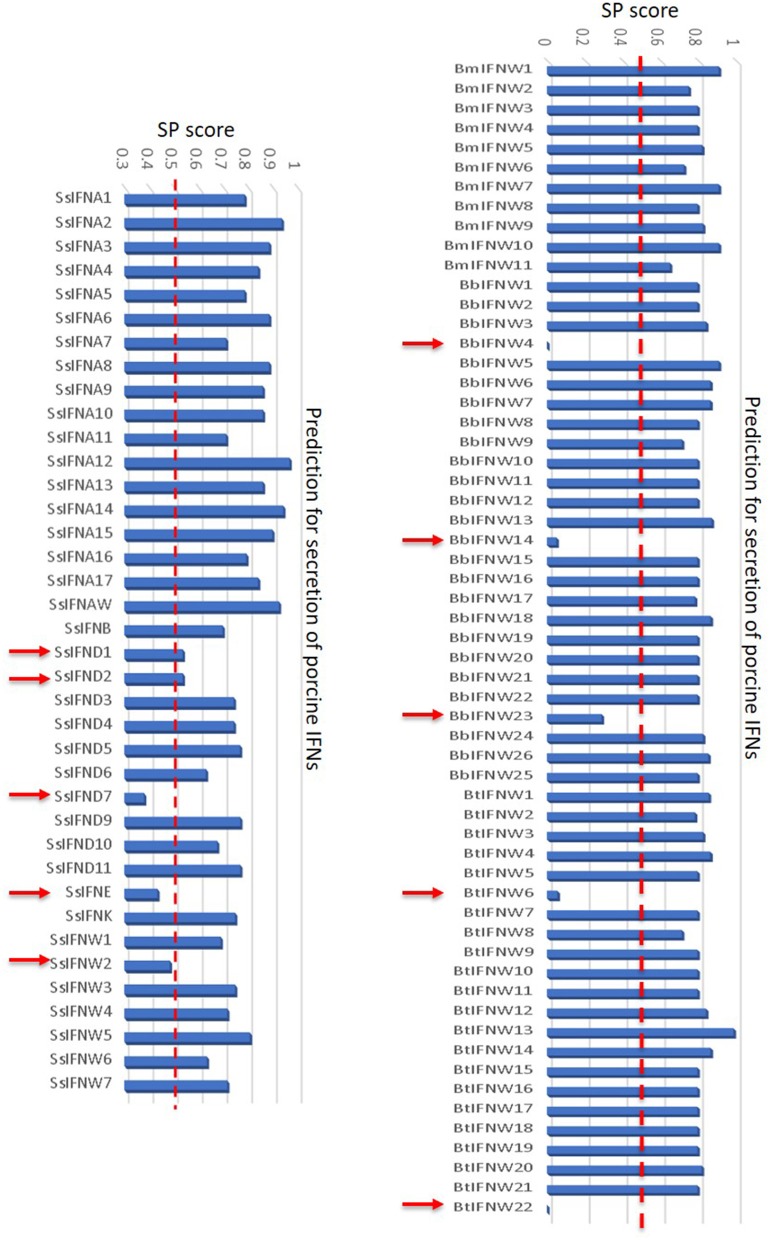
Signal peptides of swine and bovine IFN peptides were examined using PrediSi (http://www.predisi.de) to determine the secretory potency of relevant IFN mature peptides, indicating the evolution of intracellular IFNs (indicated by arrows, signal peptide prediction score 0–0.5) in each subgroup, particularly of unconventional IFN subtypes such as IFN-δ and IFN-ω subtypes that undergoing multi-gene expansion such as in pigs and cattle (2, 9).

In addition to the major clusters of the unconventional IFN subtypes in livestock, we also detected some “outliers” per molecular signatures. For example, porcine IFN-δ1, -δ2, and -ω2, as well as bovine IFN-ω4 (Bb), -ω6 (Bt), -ω14 (Bb), -ω22 (Bt) show less sequence identity (<80%) overall to other members of the same IFN subtypes (Figures 2, 4). As these members generally showed little antiviral activity in all our tests (9, 13, 23), we also detected that these “outliers” of the unconventional IFN subtypes generally do not contain signal peptides for extracellular secretion of the mature IFN peptides (Figure 10) (10, 37). This indicates that these “outliers” may represent a group of IFNs that mediate IFN responses via a newly identified intracellular pathway (10, 37), or a non-canonical IFN signaling pathway to mediate some unknown functions (10, 37–39).

## Discussion

Based on fragmentary information, previous studies on IFN molecular evolution posited a general linear increase of IFN-coding genes during vertebrate evolution and a dramatic acceleration after the emergence of intronless IFN genes, presumably in reptiles (12, 40). We have examined IFN genes across the genome sequences of more than 110 animal species, and specifically characterized the emergence and expansion of intronless IFNs in amphibians (Figure 1) (10, 12). Further subtype-diversification of intronless type I IFNs in ungulates, especially in livestock species including pigs and cattle, comprises other evolutionary surges of IFN gene expansions (8, 9). Swine and bovine species thus contain a large gene number of IFNs including several multi-gene IFN subtypes such as IFN-δ, -ω, and –τ, which represent an apex of IFN gene expansion in mammalian species (1–9). In contrast to the surges of IFN genes in amphibians, chickens, bats, mice, and especially in pigs and cattle, we also observed intriguing reduction of IFN genes in wild birds and underground rodent species (such as naked mole-rats) (Figure 1). These findings support a species/lineage-independent “bouncing” model of IFN molecular evolution and subtype-diversification across vertebrates (2, 10, 12, 40). The porcine (and bovine) IFN complex thus symbolizes a significant surge in IFN molecular evolution, which is distinguished by the expansion of multi-gene IFN subtypes beyond the classical IFN-α subtype (Figure 1) (8, 9). As the emergence and rapid expansion of intronless IFNs in amphibians were ascribed to cope with dramatic environmental changes during terrestrial adaption (10, 12), we interpret that most species-dependent evolution surges or retreats in IFN gene numbers are related to increased or decreased chances in pathogenic exposure (particularly intracellular ones like viruses) in their ambient habitats (1–7). The obvious IFN gene expansion such as in chickens, mice, pigs and cattle are likely relevant to their “domestic” process with humans, which are implicated by the increasing IFN gene numbers along three bovine species, i.e., wild yak (*Bos mutus*), zebu cattle (*Bos indicus*), and cows (*Bos taurus*) (Figure 1) (2, 41).

Although amphibians such as *Xenopus* have diversified 20–30 of intronless IFN molecules, these amphibian IFNs seem to have evolved independently and share little molecular phylogeny (<45%) to IFN subtypes in mammals (2, 10, 12). The early IFN-ω-like genes (also potentially to be progenitors of IFN-β or –κ) were determined in reptiles and clearly in some avian species (2). Similar to the diversification of other unconventional IFN subtypes, IFN-ω subtype is common in almost all mammalian species and rapidly evolved into multi-gene IFN subtype in ungulate, bats and some carnivore species, with the exception of rodents. Human and other primate species only have one IFN-ω molecule in each species (Figures 1, 2) (2). Human IFN-ω was demonstrated to be a leukocyte interferon, which had antiviral, anti-proliferation, and antitumor activities that are similar (but more broadly active) to those of IFN-α (20). Previous studies in feline IFN-ω explored them as a therapeutic option for some autoimmune diseases or retroviral infections in humans and other animals. Some recombinant feline IFN-ω peptides have been licensed in several countries for treating canine parvovirus, feline leukemia virus, and feline immunodeficiency virus infections (18, 42), indicating the broad antiviral potency and therapeutic potentials of this unconventional IFN subtype (13, 19, 20). Others and we have genome-wide analyzed the superior IFN complex in ungulate species especially in pigs and cattle (8, 9), indicating that these livestock species contain the IFN complex including IFN-ω subtype which may conceive distinct IFN molecules having higher antiviral or other biological activity. In addition to these genetically fixed IFN alleles, we have also identified many polymorphic isoforms across the porcine type I IFN gene family (14, 29), of which some differ by only one or a few residues but exert dramatic antiviral alterations, such as by different IFN-ω variants (Figure 3) (14, 29). Indeed, such as those among seven porcine IFN-ω5 isoforms, we determined a polymorphism (IFN-ω5-2, Figure 3) that has much higher antiviral activity broadly in all our analyses including seven different virus-cell systems (Figure 9) (14, 19). This correlates with the seminal discovery of structure-activity relationship of IFN site-mutants by Thomas et al. (29) and indicates the efficacy of “fine-tuning” approaches in optimization of IFN biology activity (29, 43).

Both species-specific and cross-species activities of IFNs have been reported previously and observed in our studies (2, 18, 20, 42). Theoretically, IFN activity is determined by the compatibility of an IFN ligand with cell IFN receptors. Due to the evolution of both IFNs and IFN receptors across the vertebrates, typical IFN subtypes such as IFN-α/β show more or less cross-species antiviral activity within each vertebrate groups such as fish, amphibians, reptiles, birds, and mammals. However, the cross-species activity is rare between species of two groups (unpublished data). In contrast, cross-species activity of species-specific IFN subtypes (such as IFN-δ/τ/ξ in pigs, cattle, and mice, respectively) should be limited due to phylogenic distinctness. Whereby, mammalian IFN-ω subtype seems to retain at least antiviral activity within most mammalian species as demonstrated with the single human IFN-ω as well as multiple IFN-ω peptides in cats and pigs (2, 18, 20, 42). In summary, both species-specific and cross-species IFN activity are two sides of the same coin, which reflects the variation and conservation during IFN evolution (2, 4, 7, 20).

Both phylogenic and cluster analyses of cross-species IFN-ω molecules at protein levels imply that mammalian IFN-ω subtype was diverted from a common IFN ancestor gene during the evolution of reptiles or birds (2). Even though mammalian IFN-ω subtype is similar to its IFN-α orthologs with regard to the antiviral or other biological activity, we show that its molecular origin may be closer to other common IFN subtypes such as IFN-ε/κ. After subtype-ramification, IFN-ω seems further diversify independently in different mammalian Family/Genus. However, cross-species analysis of IFN-ω sequence similarity at the protein level demonstrated that there are two general IFN-ω subgroups existing in such as moles, bats, shrews, and elephants, indicating IFN-ω molecules in these mammalian species might be derived from two close progenitors or further ramified along two directions (2, 41). In contrast, IFN-ω molecules in pigs and cattle were generally clustered into one big cluster with several outliers presumably derived from a recent gene recombination event that may be catalyzed by some genetic repetitive elements as demonstrated in previous studies (Figure 4) (2, 8–10).

Although human IFN-ω was previously determined as a leukocyte cytokine (20), our expression assays indicate that the multiple genes of porcine IFN-ω subtype are differentially expressed in different tissues/cells, and that their expression could be in either constitutive or an induced manner by a viral infection. In general, the expression patterns of porcine IFN-ω genes liken to unconventional IFN subtypes such as IFN-ε/κ/δ than the IFN-α subtype in the constitutive situation (Figure 5A) (9, 13); however, the porcine IFN-ω genes showed their own pattern of induced expression during viral infection. Notwithstanding, the fact that more gene-specific assays to examine their diverse expression at both RNA and protein levels are needed. The current data imply the necessity for gene-specific (at least subtype-specific) expression and activity analysis for IFN-ω subtype, especially in the animal species such as pigs, bats, and cattle, where they show multi-gene expansion of IFN-ω subtype (13, 24).

Compared with the classical IFN-α subtype, the antiviral activity of porcine IFN-ω peptides (especially IFN IFN-ω5) showed similar acidic stability but higher resistance to heat treatments (Table 1). Increased thermostability is correlated to the increase in the number of hydrogen bonds and in polar surface area fraction of a protein (44). We interpret that the thermal stability of some IFN-ω peptides may reflect their property in tertiary structure, which in turn may contribute to the broader and higher antiviral activity by the affinity of the IFN ligand-receptor interaction (29). Currently, there are few, if any, studies comparing the affinity difference between IFN-ω and IFN-α/β to the common IFN receptors of IFNAR1/2 (29).

In the canonical IFN signaling, IFNs engage type-specific IFN receptors (such as IFNAR1/2 or IFNLR1/IL10RB for type I or III IFNs, respectively) on the cytoplasmic membrane to induce expression of hundreds of ISGs that play roles in restricting viruses and regulating other biological responses (4, 38). To compare the efficacy of IFN-ω in induction of ISGs with IFN-α/β, we detected the expression of six ISGs representing both robust antiviral genes and tunable immunomodulatory genes across porcine, human, monkey and mouse cells (35, 41). Porcine IFN-ω subtype, particularly IFN-ω5, generally showed higher activity in induction of some robust genes and most tunable genes, as well as being broader efficacy in human, monkey, and mouse cells. We hypothesize that this differential activity in induction of ISGs contributes to the superior antiviral activity of the IFN-ω5 peptide; however, which ISGs play a critical role will require future mechanistic studies using the knock-out/-in models (35, 41). In contrast to the pro-inflammatory role of IFN-γ in immune regulation, recent studies demonstrated primary anti-inflammatory activity as well as immune-homeostatic regulation of innate immune IFNs (3, 4). In this regard, we and others have shown that unconventional IFNs, such as porcine IFN-ω and –δ subtypes, may have evolved to be subject for anti-inflammatory regulation during antiviral response, particularly for those IFN subtypes that show constitutive and epithelial expression (1–7).

The majority of IFN studies have concentrated on the induced expression of classical antiviral IFN-α/β responding to viral infection or treatments with viral mimics (1–7). Recent studies highlighted the tissue-specific expression of unconventional IFN subtypes such as IFN-λ in gut epithelia, IFN-κ in skin, and IFN-ε/τ in reproductive tract (5, 15, 31, 32). Thus, more gene-specific expression analyses need to be developed to determine potential tissue- or even cell-specific expression of the expanding unconventional IFN subtypes (such as IFN-δ/τ/ω in pigs and cattle) to elucidate functional characterization of these less-studied IFN subtypes. In addition, recent studies of the multi-functional property of unconventional IFNs also propose that IFNs can mediate immune or other physiological responses via non-canonical signaling pathways including that by intracellular IFN-signaling independent of membrane-bound IFNAR and through MAPK- and PI3K-mediated pathways independent to STAT transcription factors (4, 23, 37–41). Currently, direct evidence to determine if some IFN-ω may adopt these non-canonical signaling pathways to mediate subtype-specific IFN responses is lacking; however, our discovery of their differential biological responses in antiviral, anti-proliferation, and ISG induction, especially the lack of a signal peptide for extracellular secretion, all imply this possibility (4, 23, 37–41).

In summary, genome-wide annotation of the IFN complex across more than 110 representative species of vertebrates allows us to extensively re-examine the IFN evolutionary model (2, 10, 40), which serves as a critical molecular marker for immune evolution simultaneous to emergence of adaptive immunity in vertebrates (45). Our extensive phylogenic analyses refined several “turning-points” in IFN evolution, including the emergence and expansion of intronless IFN genes in amphibians as well as further IFN-subtype diversification surge in bats and especially in ungulate species (2, 8–12). Furthermore, we emphasize the necessity for molecular and functional characterization of unconventional IFN subtypes using several animal models, which show significant IFN expansion thus providing molecular resource for identifying and optimizing “super” IFNs for therapeutic application in either antiviral or immunomodulatory directions (3, 18, 19). We propose using the porcine IFN model, that unconventional IFN subtypes such as IFN-ω are promising for developing IFN-based antivirals that exert antiviral activity superior to classical IFN-α/β subtypes, which stresses the multi-functional property of IFN cytokines beyond the several well-studied subtypes and calls for mechanistic studies of the non-canonical IFN signaling pathways (4, 37–41).

## Data Availability

The datasets generated for this study can be found in NIH Short Read Archive linked to a BioProject with an accession number of SRP033717.

## Author Contributions

YS conceived and designed the study, interpreted data, and conformed the manuscript. FB, WM, and LM assisted in conception and design as well as for critically reading-proof. YS and WM supervised the students for data collection. LS, JJ, QL, and JL conducted experiments, data analysis, and proofreading of the manuscript.

### Conflict of Interest Statement

The authors declare that the research was conducted in the absence of any commercial or financial relationships that could be construed as a potential conflict of interest.
